# Plasma MicroRNA Panel for Minimally Invasive Detection of Breast Cancer

**DOI:** 10.1371/journal.pone.0076729

**Published:** 2013-10-23

**Authors:** Katarina Cuk, Manuela Zucknick, Dharanija Madhavan, Sarah Schott, Michael Golatta, Jörg Heil, Frederik Marmé, Andrey Turchinovich, Peter Sinn, Christof Sohn, Hans Junkermann, Andreas Schneeweiss, Barbara Burwinkel

**Affiliations:** 1 Molecular Epidemiology, German Cancer Research Center (DKFZ), Heidelberg, Germany; 2 Molecular Biology of Breast Cancer, University Women’s Clinic, Heidelberg, Germany; 3 Division of Biostatistics, German Cancer Research Center (DKFZ), Heidelberg, Germany; 4 Department of Gynecology and Obstetrics, University Women’s Clinic, Heidelberg, Germany; 5 National Center for Tumor Diseases (NCT), Heidelberg, Germany; 6 Institute of Pathology, University Hospital Heidelberg, Heidelberg, Germany; Dartmouth, United States of America

## Abstract

Over the last few years, circulating microRNAs (miRNAs) have emerged as promising novel and minimally invasive markers for various diseases, including cancer. We already showed that certain miRNAs are deregulated in the plasma of breast cancer patients when compared to healthy women. Herein we have further explored their potential to serve as breast cancer early detection markers in blood plasma. Circulating miR-127-3p, miR-376a and miR-652, selected as candidates from a miRNA array-based screening, were found to be associated with breast cancer for the first time (n = 417). Further we validated our previously reported circulating miRNAs (miR-148b, miR-376c, miR-409-3p and miR-801) in an independent cohort (n = 210) as elevated in the plasma of breast cancer patients compared to healthy women. We described, for the first time in breast cancer, an over-representation of deregulated miRNAs (miR-127-3p, miR-376a, miR-376c and miR-409-3p) originating from the chromosome 14q32 region. The inclusion of patients with benign breast tumors enabled the observation that miR-148b, miR-652 and miR-801 levels are even elevated in the plasma of women with benign tumors when compared to healthy controls. Furthermore, an analysis of samples stratified by cancer stage demonstrated that miR-127-3p, miR-148b, miR-409-3p, miR-652 and miR-801 can detect also stage I or stage II breast cancer thus making them attractive candidates for early detection. Finally, ROC curve analysis showed that a panel of these seven circulating miRNAs has substantial diagnostic potential with an AUC of 0.81 for the detection of benign and malignant breast tumors, which further increased to 0.86 in younger women (up to 50 years of age).

## Introduction

Breast cancer is the most common type of cancer and the leading cause of cancer-related death among women in industrialized countries. Worldwide approximately 1.3 million women develop breast cancer each year [1]. Fortunately, over the years there has been a decline in mortality rates, which can be attributed to the advances made in early diagnosis and treatment [1]. Nevertheless, tens of thousands of women die from breast cancer each year. Early detection is important as the overall 5-year survival is >90% when diagnosed at an early stage as opposed to ∼20% when the disease has already spread to distant organs [2].

Mammography is the standard breast cancer screening tool, but there are controversial reports regarding its utility as a screening method [3], [4]. Furthermore, mammographic screening seems to be less sensitive for younger women, possibly due to an increased mammographic breast density, which is usually associated with younger age [5], [6]. As of now there are no circulating markers for breast cancer screening or detection in clinical use, but a few such markers, e.g. carcinoembryonic antigen (CEA) or carbohydrate antigen 15-3 (CA 15-3), are being used and seem helpful for making decisions in the metastatic setting [7].

MicroRNAs (miRNAs) are a class of small, non-coding RNAs (∼22 nucleotides in length) that regulate gene expression on a post-transcriptional level [8]. By degrading mRNA molecules or blocking their translation miRNAs play an essential role in the regulation of a large number of biological processes, including cancer [9]. In 2008 first reports emerged demonstrating the presence of circulating miRNAs in cell-free body fluids such as plasma and serum [10], [11]. Since then, circulating miRNAs have been reported as being deregulated in blood plasma or serum in different types of disease, including cancer [12], [13]. Although the origin of circulating miRNAs is heterogeneous and still under debate [14], the possibility of their repeated measurement in a minimally invasive manner as well as their remarkable stability in plasma/serum make them attractive candidates for the development of novel markers [15], [16].

The aims of this study were to improve the breast cancer detection capability by investigating additional miRNA marker candidates, which we selected from a miRNA array-based approach, and to independently validate our previously identified breast cancer associated miRNAs. We linked circulating miR-127-3p, miR-376a and miR-652 to breast cancer for the first time and independently validated them, together with miR-148b, miR-376c, miR-409-3p and miR-801, as elevated in the plasma of breast cancer patients in a second study cohort. Finally, a combination of these seven circulating miRNAs represents our diagnostic miRNA panel for discriminating between healthy women and patients with benign and malignant breast tumors, with superior performance in younger women.

## Materials and Methods

### Breast Cancer Patients and Healthy Controls

This study was approved by the Ethical Committee of the Medical Faculty in Heidelberg. Two study cohorts were investigated. In study cohort A (n = 207) blood samples were collected from 127 sporadic breast cancer patients and 80 healthy female volunteers, who served as controls. In study cohort B (n = 210) samples were collected from 30 women with benign and 120 with malignant breast tumors, respectively, as well as 60 healthy female individuals. All cases and controls were Caucasian. After giving written informed consent patient blood samples were collected between 2010 and 2012 at the time-point of diagnosis before they underwent any therapeutic treatment, such as surgery, radiation or systemic therapy. Patient clinico-pathological features were defined by operative findings. For neoadjuvant patients (n = 26 in cohort A and n = 24 in cohort B), histo-pathological characteristics and tumor stage were assessed based on histobiopsy results and imaging techniques. Control blood samples were collected between 2010 and 2012 from healthy women with no history of malignant diseases, no blood transfusions received in the previous 3 years and no current inflammatory condition (based on self-report). Table 1 and Table S1 summarize the clinical features of the patients and lifestyle data of the healthy controls, respectively. No clinico-pathological data is available for the women with benign breast lesions, but their mean and median ages were 47 and 46 years (age range: 32–71 years), respectively. Tissue sample harvesting and clinico-pathological characteristics are described in [12].

**Table 1:** Clinico-pathological characteristics of the malignant breast cancer patients in validation cohorts A and B.

Characteristics		Cohort A(n = 127)	Cohort B (n = 120)
**Age range (years):**		31–83	30–77
**Mean (median) age:**		56.6 (55.0)	48.6 (48.0)
**Menopausal status**	premenopausal	43	59
	perimenopausal	9	14
	postmenopausal	73	47
	unknown	2	0
**ER status** *	positive	103	99
	negative	24	21
**PR status** *	positive	96	88
	negative	31	30
	unknown	0	2
**HER2 status** **	positive	14	25
	negative	113	95
**Histological tumor** **grade**	1	12	13
	2	72	79
	3	41	26
	unknown	2	2
**p53 tumor marker**	0%	11	6
	≤10%	79	55
	11–20%	1	2
	21–30%	2	3
	>30%	20	19
	unknown	14	35
**Ki-67 proliferation marker**	0%	0	1
	≤10%	46	36
	11–20%	18	33
	21–30%	11	14
	>30%	24	18
	unknown	28	18
**Histological subtype**	IDC	101	101
	ILC	11	8
	IDC/ILC	3	0
	unknown	12	11
**Tumor size**	*in situ*	4	0
	pT1	47	51
	pT2	60	53
	pT3	10	13
	pT4	2	3
	unknown	4	0
**Lymph nodes**	N0	75	71
	N1	34	33
	N2	6	9
	N3	6	1
	unknown	6	6
**Metastasis**	M0	116	118
	M1	9	2
**Tumor stage (AJCC)**	0	4	0
	I	38	39
	II	58	55
	III	14	22
	IV	9	2
	unknown	4	2
**Tumor focality**	unifocal	83	85
	multiple foci	37	24
	unknown	7	11

ER = estrogen receptor; PR = progesterone receptor; HER2 =  human epidermal growth factor receptor 2; IDC = invasive ductal carcinoma; ILC = inv. lobular carcinoma.

* immunoreactive score (IRS): 0–2 =  ER/PR negative; 3–12 =  ER/PR positive.

** IHC-score: 0–1 =  HER2 negative; 2 =  positive if HER2 amplified in FISH/CISH; 3 =  HER2 positive.

### Blood Processing and miRNA Extraction from Plasma

EDTA blood samples were processed for plasma within 2 hours of collection. Blood was centrifuged at 1300 g for 20 minutes at 10°C followed by a second high-speed centrifugation step of the supernatant (plasma) at 15500 g for 10 minutes at 10°C to remove cell debris and fragments. The plasma was snap-frozen in liquid nitrogen and stored at −80°C until use. Total RNA (including miRNAs) was extracted from 400 µL of plasma as described in [12].

### miRNA Quantification and Statistical Analysis

Reverse transcription (RT) reactions in plasma and tissue samples were performed using TaqMan miRNA Reverse Transcription Kit (Applied Biosystems, Germany) and miRNA-specific RT primers for hsa-miR-127-3p, hsa-miR-148b, hsa-miR-376a, hsa-miR-376c, hsa-miR-409-3p, hsa-miR-652, hsa-miR-801 and cel-miR-39 (Applied Biosystems, Germany). RT reactions and TaqMan real-time PCR were carried out in a blinded manner as previously described in [12].

Raw miRNA data was normalized to spiked-in cel-miR-39 as described in [12]. Statistical analysis was performed using the computational environment R version 2.11 and 2.13 (http://www.r-project.org/) [17]. Briefly, Wilcoxon rank sum tests with continuity correction were used to identify deregulated miRNAs. For associations between miRNA levels and clinico-pathological (breast cancer cases) or lifestyle data (healthy controls) following non-parametric tests were used: Wilcoxon rank sum test (binary categorical variables), Spearman’s rank correlation test (continuous variables) and Jonckheere-Terpstra test (ordinal variables). A two-tailed P<0.05 was considered statistically significant. Breast cancer detection potential was evaluated by computing receiver operating characteristic (ROC) curves and calculating areas under the curves (AUC) as well as specificities for fixed sensitivity values with corresponding 95% confidence intervals (CI) [18], [19]. Inter-relationships between miRNA levels were investigated by computing Spearman rank correlation coefficients (ρ). Tissue sample analysis was performed as previously described in [12].

## Results

### New miRNA Candidates (miR-127-3p, miR-376a and miR-652) are Present at Higher Levels in the Plasma of Breast Cancer Patients

In our previous work, we have conducted an initial screening using TaqMan Low Density (TLDA) arrays to identify circulating miRNAs deregulated between early stage breast cancer patients and healthy controls (Table S2 and [12]). In order to improve our breast cancer detection accuracy here we re-analyzed the TLDA data using less stringent analysis criteria, i.e. by omitting the removal of miRNAs with low inter-quartile ranges (IQR) after quantile normalization (Table S3). Subsequently, three additional miRNA candidates (miR-127-3p, miR-376a and miR-652) were selected for validation studies after applying the following criteria: (i) P<0.05, (ii) mean Ct<33 in at least one investigated group and (iii) |ΔCt|>1 (indicating that the miRNA amounts in the patients and controls differ markedly).

**Figure 1:**
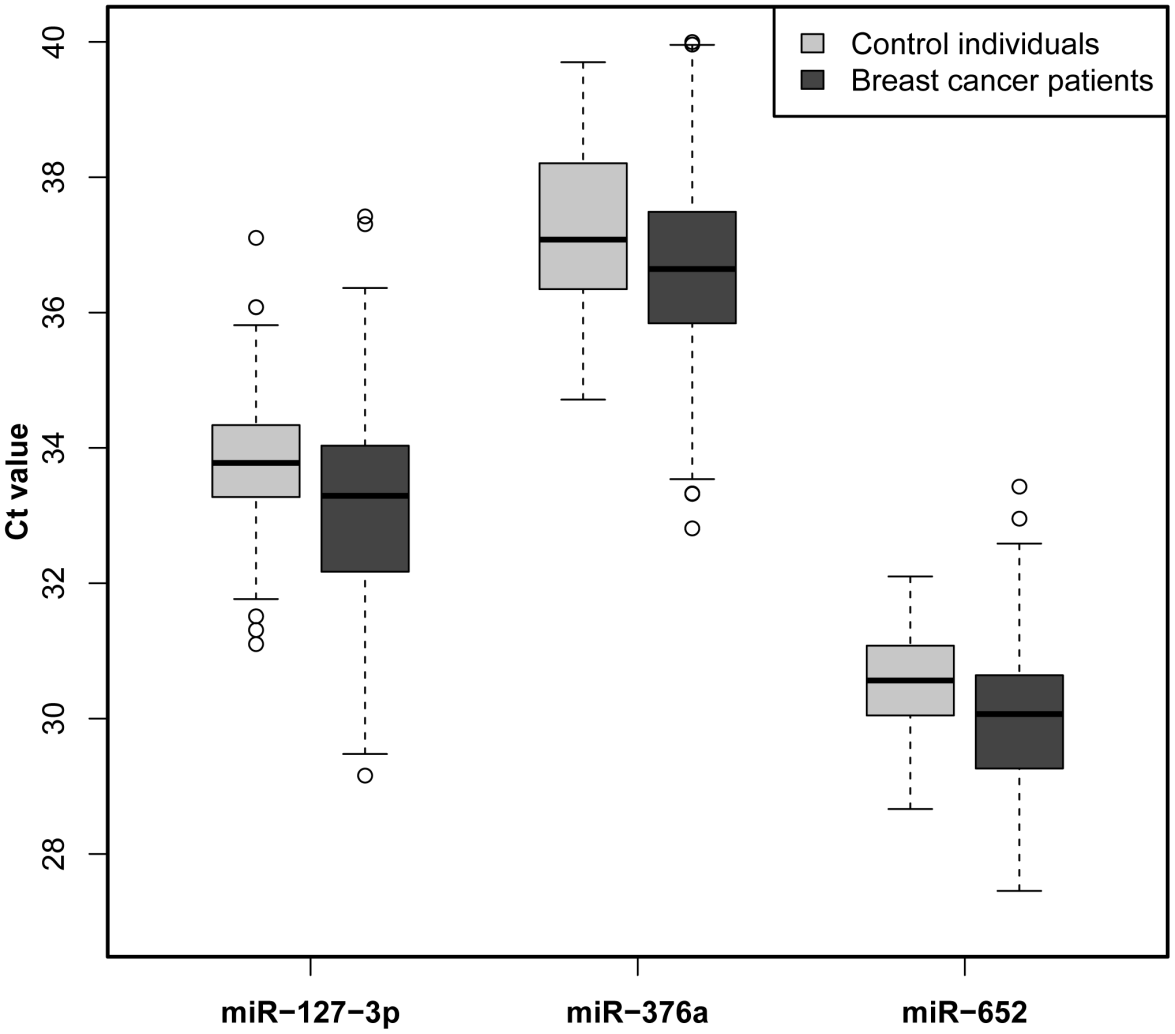
Investigation of three new miRNA marker candidates (miR-127-3p, miR-376a and miR-652) in cohort A. Circulating miR-127-3p, miR-376a and miR-652 are present at elevated levels in the plasma of breast cancer patients when compared to healthy women. A two-tailed P<0.05 was considered significant (Wilcoxon rank sum test).

First we measured the miRNA levels of miR-127-3p, miR-376a and miR-652 in study cohort A (n = 207), comprising a total of 127 breast cancer patients and 80 healthy controls (Table 1 and Table S1), and found that miR-127-3p (P<0.001), miR-376a (P = 0.01) and miR-652 (P<0.0001) are present at higher levels in the plasma of breast cancer patients when compared with healthy women (Figure 1 and S1). Next we expanded our analysis on the independent validation cohort B (n = 210).

### Seven Circulating miRNAs were Independently Validated as Elevated in the Plasma of Breast Cancer Patients

**Figure 2:**
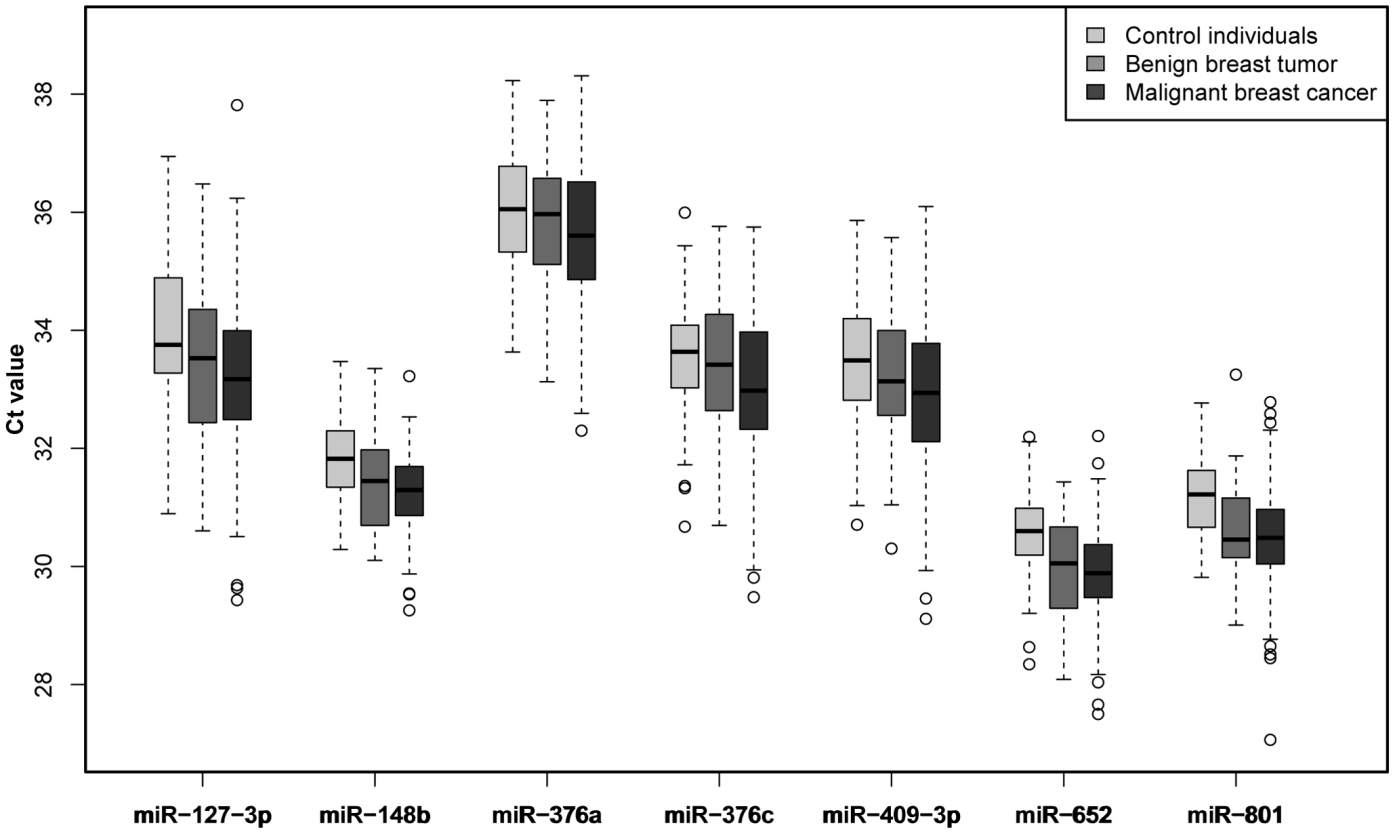
Independent validation of seven circulating miRNAs (cohort B). Circulating miR-127-3p, miR-148b, miR-376a, miR-376c, miR-409-3p, miR-652 and miR-801 levels were independently validated as being elevated in the plasma of malignant breast cancer patients compared to healthy women. Circulating miR-148b, miR-652 and miR-801 were also elevated in the plasma of women with benign breast tumors when compared to healthy individuals. A two-tailed P<0.05 was considered significant (Wilcoxon rank sum test).

By investigating an independent validation cohort, we confirmed that circulating miR-127-3p (P = 0.0003), miR-148b (P<0.0001), miR-376a (P = 0.03), miR-376c (P = 0.03), miR-409-3p (P = 0.005), miR-652 (P<0.0001) and miR-801 (P<0.0001) are increased in the plasma of women with breast cancer when compared to healthy controls (Figure 2). Also, we found that miR-148b (P = 0.02), miR-652 (P = 0.01) and miR-801 (P = 0.003) differ significantly even in the plasma of women with benign breast lesions when compared to healthy women (Figure 2). By stratifying the malignant breast cancer patients according to their cancer stage, we observed that miR-127-3p, miR-148b, miR-409-3p, miR-652 and miR-801 are able to identify even stage I or stage II cancer patients when compared to healthy controls (Table S4).

Additionally, we noticed that miR-127-3p, miR-376a, miR-376c and miR-409-3p belong to the same miRNA cluster, which is located on the chromosome region 14q32. This finding implies an over-representation of chromosome 14q32 region miRNAs as being deregulated in the plasma of breast cancer patients.

### Correlation of Plasma miRNA Levels to Clinico-pathological and Lifestyle Data

Table 1 and Table S1 summarize the clinico-pathological features (patients) and lifestyle data (controls) of study cohort B, which were used for correlation with miRNA levels. In the breast cancer samples miR-801 levels displayed correlations to age (P = 0.04), menopausal status (P = 0.01), tumor grading (P = 0.0005), progesterone receptor status (P = 0.02), the tumor marker p53 (P = 0.04) and the proliferation marker Ki-67 (P = 0.01), while miR-148b (P = 0.007) and miR-652 (P = 0.0005) correlated with the tumor marker p53.

In the control samples miR-127-3p levels correlated with age of menarche (P = 0.02) and parity (P = 0.006), when comparing women who were never pregnant with those who were. Circulating miR-376a and miR-376c also showed correlations with the parity (P = 0.007 and P = 0.04, respectively). Finally, miR-801 levels correlated with age (P = 0.04) in the control group as well (Figure S2).

### Diagnostic Potential of Circulating miRNAs

Prior to analyzing the diagnostic potential we analyzed the inter-correlations between the seven circulating miRNAs in study cohort B (data from the first validation cohort A is not shown, but was comparable). We found that the levels of miR-127-3p, miR-376a, miR-376c and miR-409-3p correlate strongly to each other with Spearman rank correlation coefficients ρ>0.80 (Table S5). Additionally, circulating miR-148b correlated considerably with miR-127-3p (ρ = 0.62) and miR-652 (ρ = 0.78), whereas miR-801 showed no significant correlations except for a slight correlation to miR-148b levels (ρ = 0.24).

The diagnostic potential of circulating miRNAs was evaluated by ROC curve analysis and the discriminatory accuracy presented by AUC values. In the herein analyzed cohort B women with benign breast tumors were included to investigate whether the circulating miRNAs can detect benign breast lesions as well. In brief, a combination of these seven circulating miRNAs yielded a good discriminatory accuracy (AUC = 0.81; 95% CI = 0.72–0.91) for benign breast tumors (Figure S3). Furthermore, the accuracy was higher in younger women up to the age of 50 years (AUC = 0.87; 95% CI = 0.77–0.97). The discriminatory accuracy for differentiating between malignant breast cancer patients and healthy controls was the highest for a combination of all seven miRNAs with AUC = 0.81 (95% CI: 0.75–0.88) and an even better AUC of 0.85 (95% CI: 0.78–0.93) for younger women only (Figure S4).

**Figure 3:**
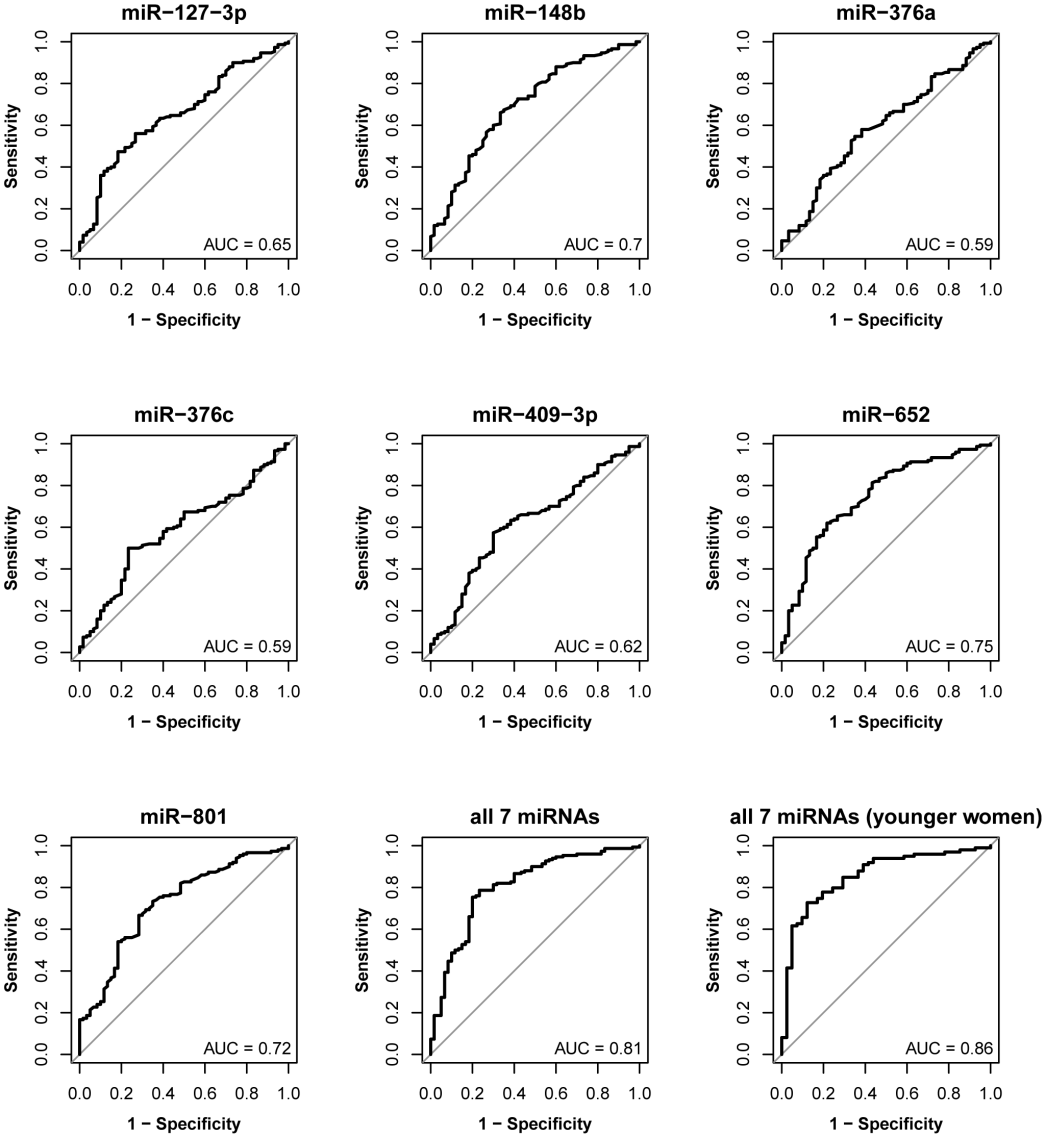
Diagnostic potential of deregulated circulating miRNAs for benign and malignant breast tumors (cohort B). In ROC curve analysis individual circulating miRNAs were found to have discriminatory accuracy of 0.59–0.75. A panel of seven circulating miRNAs (miR-127-3p, miR-148b, miR-376a, miR-376c, miR-409-3p, miR-652 and miR-801) discriminated between healthy women and those with benign and malignant breast tumors with an AUC of 0.81 and the discriminatory power was superior in younger women (AUC = 0.86).

Finally, individual and combined ROC curves for discriminating samples from both benign and malignant breast tumor patients versus healthy women are shown in Figure 3. Here, circulating miR-127-3p had an AUC of 0.65 (95% CI: 0.57–0.73), miR-148b of 0.70 (95% CI: 0.62–0.78), miR-376a of 0.59 (95% CI: 0.51–0.67), miR-376c of 0.59 (95% CI: 0.51–0.67), miR-409-3p of 0.62 (95% CI: 0.54–0.70), miR-652 of 0.75 (95% CI: 0.67–0.82) and miR-801 of 0.72 (95% CI: 0.65–0.80). The combination of all seven circulating miRNAs displayed the highest discriminatory power with AUC = 0.81 (95% CI: 0.74–0.87). At a sensitivity of 80% the median specificity was 72%. Again, in younger women (up to the age of 50 years) these circulating miRNAs performed superiorly and had an even higher accuracy (AUC = 0.86; 95% CI = 0.79–0.93) for the detection of both benign and malignant breast lesions.

### miR-127-3p, miR-376a and miR-652 are Present at Lower Levels in Malignant Primary Breast Cancer Tissue

**Figure 4:**
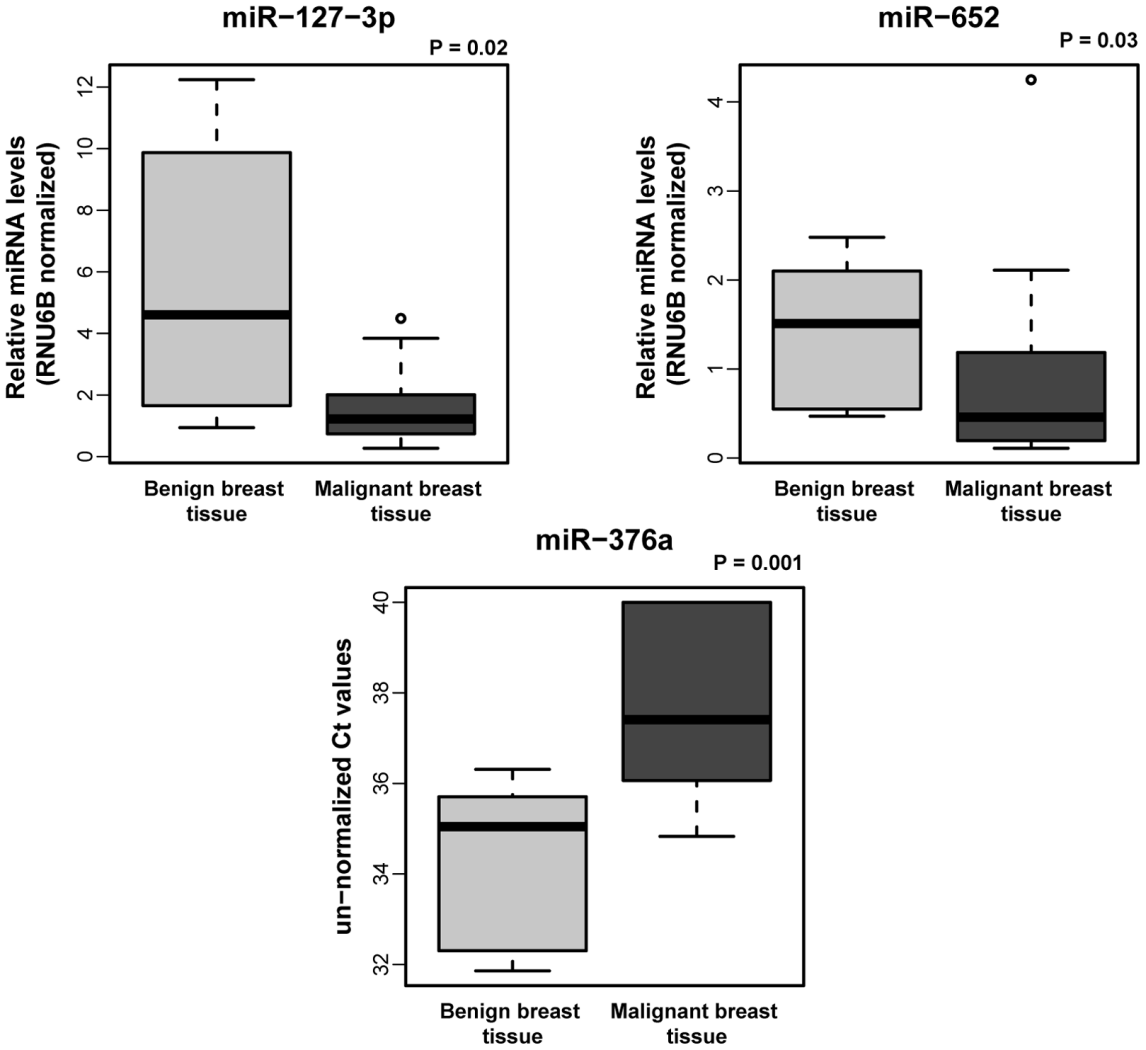
MiRNA levels in benign and malignant breast tissue. Levels of miR-127-3p, miR-376a and miR-652 are decreased in malignant primary breast cancer when compared to benign breast tissue. Box and whisker plots show RNU6B normalized relative miRNA levels for miR-127-3p and miR-652. As an exception un-normalized Ct values are presented for miR-376a as the normalization strategy was not applicable for this particular miRNA (due to the rather low miR-376a levels in the investigated tissue samples). A two-tailed P<0.05 was considered significant (Wilcoxon rank sum test).

Analysis of miRNA levels in tissue showed that miR-127-3p, miR-376a and miR-652 are present at lower levels in malignant breast cancer tissue in comparison to benign breast tissue samples (all P<0.05) (Figure 4). In our previous work we have shown that miR-148b, miR-376c and miR-409-3p (but not miR-801) are also decreased in malignant compared to benign breast tissue [12]. We also investigated the correlations of miRNA levels in malignant tissue with clinico-pathological data, but found no significant associations. Nevertheless, among the analyzed patient samples there were three with unfavorable events (distant metastasis), which prompted us to look for a possible association to the tissue miRNA levels. Surprisingly, we found that those three patients with poor outcome had the highest miR-801 levels of all analyzed malignant tissue samples (not shown).

## Discussion

One of the major challenges in the fight against cancer is early detection as it holds promise to result in a more favourable disease outcome. The standard breast cancer screening tool is mammography. Some of the disadvantages of mammography include the use of ionizing radiation and patient’s discomfort during the screening, but the most important drawback might be its poorer sensitivity in younger women, where tumor growth is the fastest [5], [6]. Therefore, novel and minimally invasive techniques, which are adequately sensitive for younger women as well, might offer a valuable alternative or complement to the existing methods.

Previous studies in plasma and serum have already identified circulating miRNAs, which seem to be able to discriminate between healthy women and breast cancer patients, such as miR-155, miR-181a, miR-299-5p, miR-411 or miR-1304 [20]–[23]. Apart from finding miRNAs with diagnostic potential, some studies revealed other intriguing observations, such as differences in miRNA expression between different ethnic groups or differences in circulating miRNA signatures in localized versus metastatic breast cancer [12], [21], [24]. These and sample processing issues might be the reasons for the ostensible lack of reproducibility in published data regarding circulating miRNAs as markers for breast cancer as discussed in greater detail in [12]. The strengths of our study are (i) standardized processing of blood samples to generate plasma within two hours of collection with a two-step centrifugation protocol, (ii) carrying out validation studies in a blinded manner, and (iii) investigation of plasma samples which have been taken at the time-point of breast cancer diagnosis before the patients underwent any therapeutic treatment.

As this study focused on early detection of breast cancer our samples were enriched for patients with stage I and stage II disease, which represented more than 80% of the investigated cancer individuals in cohort B. In addition here we included a separate group of patients with benign breast lesions. Due to a re-analysis of the TLDA array data, we identified three new miRNA candidates. In the herein presented data we confirmed our previous findings (miR-148b, miR-376c, miR-409-3p and miR-801) and showed that our new miRNA candidates (miR-127-3p, miR-376a and miR-652) are also present at significantly higher levels in the plasma of breast cancer patients when compared to healthy women (Figures 1 and 2). Interestingly, miR-148b, miR-652 and miR-801 had increased levels even in the plasma of women with benign breast tumors when compared to healthy women (Figure 2). Also, by stratifying the cancer patients into different disease stage groups, we were able to demonstrate the capability of miR-127-3p, miR-148b, miR-409-3p, miR-652 and miR-801 to identify also stage I or stage II cancer patients when compared to healthy controls (Table S4). This further strengthens the idea of utilizing them as minimally invasive, blood-based markers for early detection of this disease.

To our best knowledge this is the first study to report an over-representation of chromosome 14q32 miRNAs as deregulated in plasma and tissue of breast cancer patients. This chromosomal region contains the immunoglobulin heavy chain (IGH) locus and is suspected to harbor tumor suppressor genes as it has already been described as down-regulated in some types of cancer, such as acute lymphoblastic leukemia, uterine carcinosarcoma or melanoma [25]–[27]. Our tissue analysis confirmed this as we found the 14q32 miRNAs (miR-127-3p, miR-376a, miR-376c and miR-409-3p) to be present at lower levels in malignant breast cancer in comparison to benign breast tissue samples indicating these miRNAs might indeed be tumor suppressors (Figure 4 and [12]).

The involvement of (circulating) miR-148b, miR-376c, miR-409-3p and miR-801 in breast and other types of cancer was discussed in [12]. Regarding the three newly identified miRNA candidates, as of now miR-127-3p has been described as up-regulated in a subtype of acute myeloid leukemia and colorectal cancer with KRAS mutations and decreased in osteosarcoma cell lines and in primary breast cancer tissue [28]–[31]. Interestingly, altered miR-127-3p levels were also associated with Eppstein-Barr virus (EBV) infections in Burkitt’s lymphoma patients and human papilloma virus (HPV) infections in oral carcinomas [32], [33]. Additionally, miR-127-3p seems to be up-regulated in tumor initiating cells (important for disease recurrence and metastasis) in lung carcinoma [34]. This miRNA has also been found to be responsive to methylation-based silencing in the genomic DNA of gastric carcinomas and to radiation treatment, which causes its up-regulation in primary human dermal endothelial cells thereby enhancing their radiosensitivity [35], [36]. Lastly, miR-127-3p was also identified as part of a serum miRNA signature (which also includes miR-148b) for esophageal carcinoma detection [13]. Concordant with our findings in malignant breast cancer, miR-376a was found to be down-regulated in hepatocellular carcinoma and melanoma tissue [27], [37]. Up to now miR-652 has been described as present at lower levels in lung cancer samples when compared to matching normal lung tissue [38]. Interestingly, in an investigation of a rat hepatocarcinogenesis model, increasing serum levels of seven miRNAs (including miR-652) were observed during the carcinogenesis process, but an inverse correlation of hepatic tissue and serum miR-652 levels was found [39]. This pattern of inverse correlation of tissue and serum levels is in accordance with our observations and might cause speculation that malignant cancer cells can indeed selectively release specific miRNAs [40]. In that case a selective release of miRNAs into the blood stream could cause levels of specific miRNAs to increase in the circulation and decrease in the malignant tumor cells from which they originate. Alternatively, some of the circulating miRNAs could also originate from blood cells, the stromal compartment and/or tumor microenvironment or be some kind of an immune response to the tumor and are therefore not up-regulated in the malignant tissue itself [41]–[43]. Complete elucidation of the still unclear origin of circulating miRNAs is needed to provide an explanation for these observations.

Concordant with our previous findings plasma levels of miR-801 in the independent validation cohort B correlated to age and menopausal status in the patients (Figure S2). Additionally, we detected a correlation of miR-801 to the patients’ progesterone receptor status and more interestingly to the tumor marker p53, the tumor grading and the proliferation marker Ki-67. The latter two clinico-pathological features are indicative of the proliferation potential and aggressiveness of the tumor, so it is possible that miR-801 might be a prognostic marker as well. Unfortunately, not enough time has passed since the recruitment of the patients to have meaningful prognostic information, so we could not test this hypothesis properly yet. Interestingly, in our previous studies miR-801 was the only circulating miRNA, which we found to play a role both in primary and metastatic breast cancer, suggesting that this miRNA might be important for tumor pathogenesis as well as for tumor progression [12], [24]. Circulating miR-148b and miR-652 also might have prognostic capabilities as in cohort B they correlated with the tumor marker p53, whose addition (together with Ki-67) to conventional clinico-pathological characteristics seems to be helpful in the prognostic evaluation of breast cancer [44], [45].

Benign breast tumors can give rise to *in situ* or even invasive carcinomas, therefore it is important to detect them as well [46], [47]. In the independent validation cohort the seven-miRNA panel reached the same accuracy (AUC = 0.81) in detecting women with benign lesions as in detecting women with malignant breast tumors (Figures S3 and S4). Furthermore, in an analysis including only younger women (up to the age of 50) our seven-miRNA panel performed even better and reached an AUC of 0.87 for benign tumors and 0.85 for detecting only malignant cancer (Figures S3 and S4). Finally, the detection of both benign and malignant tumors was equally accurate with an AUC of 0.81 and, again, superior in younger women with an AUC of 0.86 (Figure 3). The increase of discriminatory accuracy of the proposed seven-miRNA panel in women up to the age of 50 years is especially important considering that mammography, the current gold standard breast cancer screening and detection method, seems to be less sensitive in younger women [5], [6], [48].

In conclusion, this is the first study to associate circulating miR-127-3p, miR-376a and miR-652 with breast cancer. In addition, we have independently confirmed that miR-127-3p, miR-148b, miR-376a, miR-376c, miR-409-3p, miR-652 and miR-801 levels are elevated in the plasma of breast cancer patients. These miRNAs can differentiate even women with benign tumors, stage I or stage II breast cancer from healthy controls thereby reinforcing their utility as minimally invasive, early detection markers. The substantial accuracy of breast tumor detection makes this circulating miRNA panel a potentially useful (pre)screening tool, especially in younger women for whom mammography seems to be less sensitive. But, further studies are necessary before these findings can be translated into clinical use.

## Supporting Information

Figure S1 **Investigation of the diagnostic potential of 3 new miRNA marker candidates (miR-127-3p, miR-376a and miR-652) in the previously published cohort A.** Circulating miR-127-3p, miR-376a and miR-652 show potential to discriminate between the patients and controls as indicated in their respective ROC curves.
(TIF)

Figure S2 **Correlation of circulating miR-801 with age in the breast cancer patients and healthy controls (independent validation cohort B).** The linear regression lines in the scatterplot of Ct values for miR-801 in the investigated samples versus the age of the individuals show the correlation of miRNA levels with age in cohort B. Empirical ROC curve for miR-801 (black line) and overlayed age-adjusted ROC curve estimates for different ages showed that there is a slight increase of the discriminatory performance of miR-801 with increasing age, but it does not look like the comparison of miR-801 between cases and controls needs to be adjusted for age, as the P-value for the interaction of miR-801 and age is not significant (P = 0.72) [Smith & Thompson, *Biometrical Journal* 1996].
(TIF)

Figure S3 **The diagnostic potential of circulating miRNAs for benign breast tumors (independent validation cohort B).** In ROC curve analysis individual circulating miRNAs had discriminatory accuracy of 0.53–0.69 for plasma samples derived from healthy women and those with benign breast tumors. The accuracy was good for circulating miR-148b, miR-652 and miR-801, which were found to be significantly elevated in the plasma of women with benign breast tumors when compared to healthy women, while it was somewhat poor for the other four miRNAs. A combination of all seven circulating miRNAs yielded the highest discriminatory power for the detection of benign tumors with an AUC equal to 0.81, which was superior for younger women up to 50 years of age, where the AUC reached 0.87.
(TIF)

Figure S4 **The diagnostic potential of circulating miRNAs for malignant breast tumors (independent validation cohort B).** In ROC curve analysis individual circulating miRNAs were found to have discriminatory accuracy of 0.60–0.77 between plasma samples derived from healthy women and those with breast cancer. A combination of all seven circulating miRNAs yielded the highest discriminatory power for the detection of malignant breast tumors with an AUC equal to 0.81, which was even higher in younger women (≤50 years) with an AUC of 0.85.
(TIF)

Table S1 **Lifestyle data of healthy controls in validation cohorts A and B.**
(DOC)

Table S2 **Original data from Human MicroRNA TaqMan Low Density Arrays (TLDA).**
(XLS)

Table S3 **Circulating miRNAs deregulated in the plasma of early stage breast cancer cases compared to healthy controls in TLDA array re-analysis.** All candidate markers we analyzed are in bold, the three new marker candidates chosen for validation are underlined and the finally validated miRNAs are additionally italicized.
(DOC)

Table S4 **Circulating miRNAs deregulated in the plasma of early stage breast cancer cases (stage I and/or stage II) compared to healthy controls.** In the validation cohorts circulating miR-127-3p, miR-148b, miR-409-3p, miR-652 and miR-801 were significantly elevated even in stage I and/or stage II breast cancer patients compared to healthy controls. A two-tailed P<0.05 was considered statistically significant (Wilcoxon rank sum test). Ctrls = controls.
(DOC)

Table S5 **Inter-correlations between miRNA levels in plasma.** Spearman rank correlation coefficients (ρ) between different circulating miRNAs with their 95% confidence intervals (CI) and P values.
(DOC)
