# Supplementary figures and images for: Plasma MicroRNA Panel for Minimally Invasive Detection of Breast Cancer
Source: PLoS One. 2013 Oct 23;8(10):e76729. doi: 10.1371/journal.pone.0076729 (PMC3806790; doi:10.1371/journal.pone.0076729)

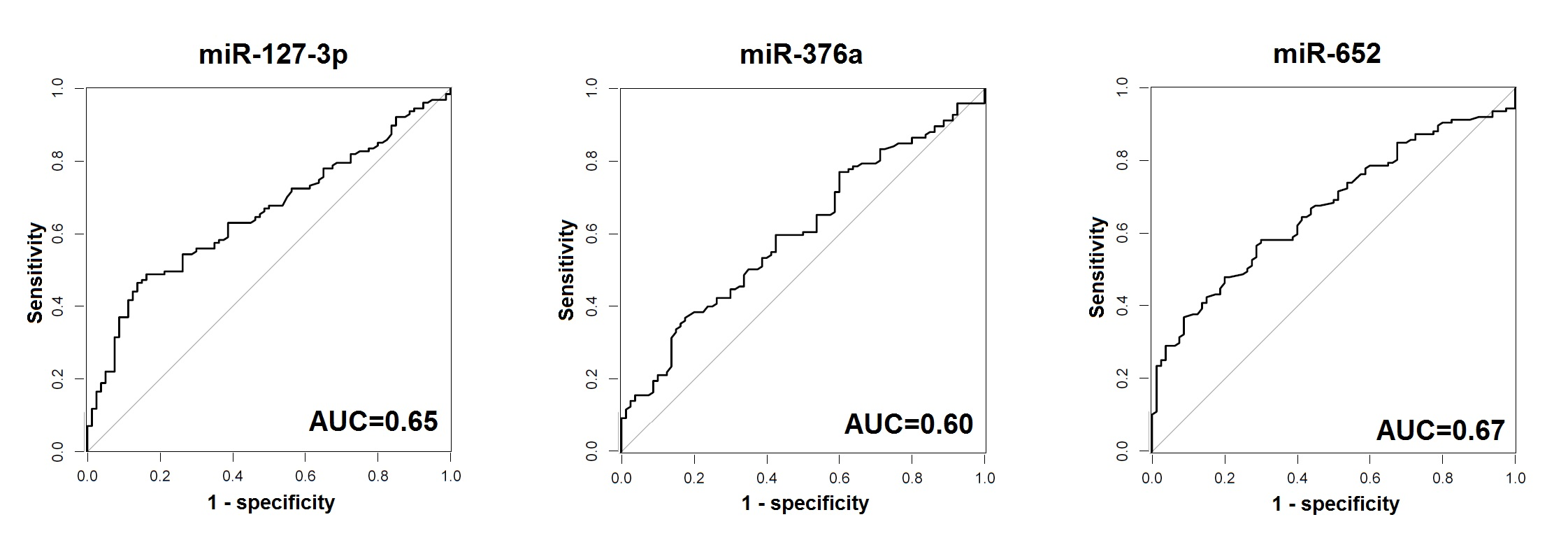

Supplement: Figure S1 — Investigation of the diagnostic potential of 3 new miRNA marker candidates (miR-127-3p, miR-376a and miR-652) in the previously published cohort A. Circulating miR-127-3p, miR-376a and miR-652 show potential to discriminate between the patients and controls as indicated in their respective ROC curves. (TIF) [file pone.0076729.s001.tif]

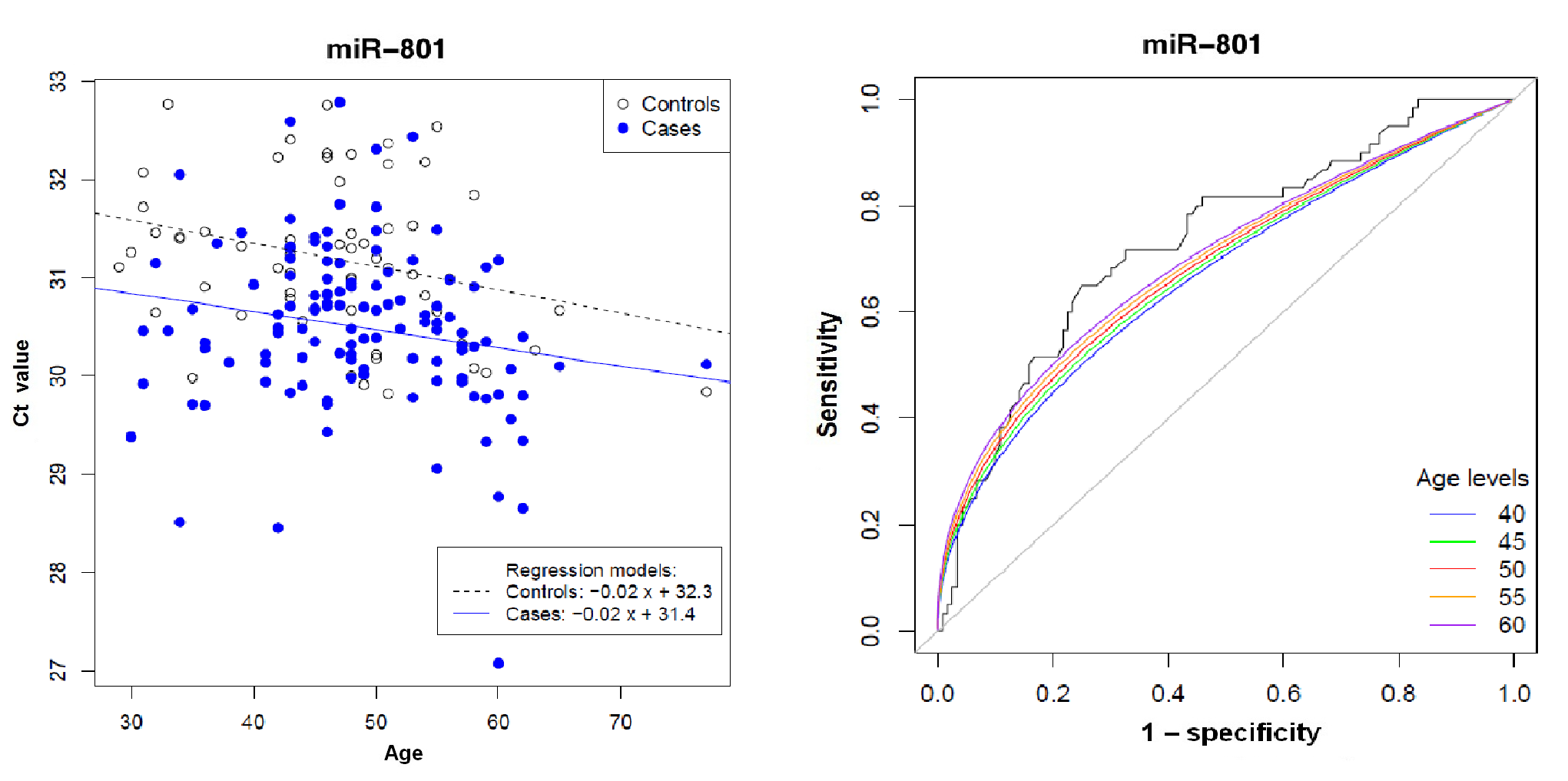

Supplement: Figure S2 — Correlation of circulating miR-801 with age in the breast cancer patients and healthy controls (independent validation cohort B). The linear regression lines in the scatterplot of Ct values for miR-801 in the investigated samples versus the age of the individuals show the correlation of miRNA levels with age in cohort B. Empirical ROC curve for miR-801 (black line) and overlayed age-adjusted ROC curve estimates for different ages showed that there is a slight increase of the discriminatory performance of miR-801 with increasing age, but it does not look like the comparison of miR-801 between cases and controls needs to be adjusted for age, as the P-value for the interaction of miR-801 and age is not significant (P = 0.72) [Smith & Thompson, Biometrical Journal 1996]. (TIF) [file pone.0076729.s002.tif]

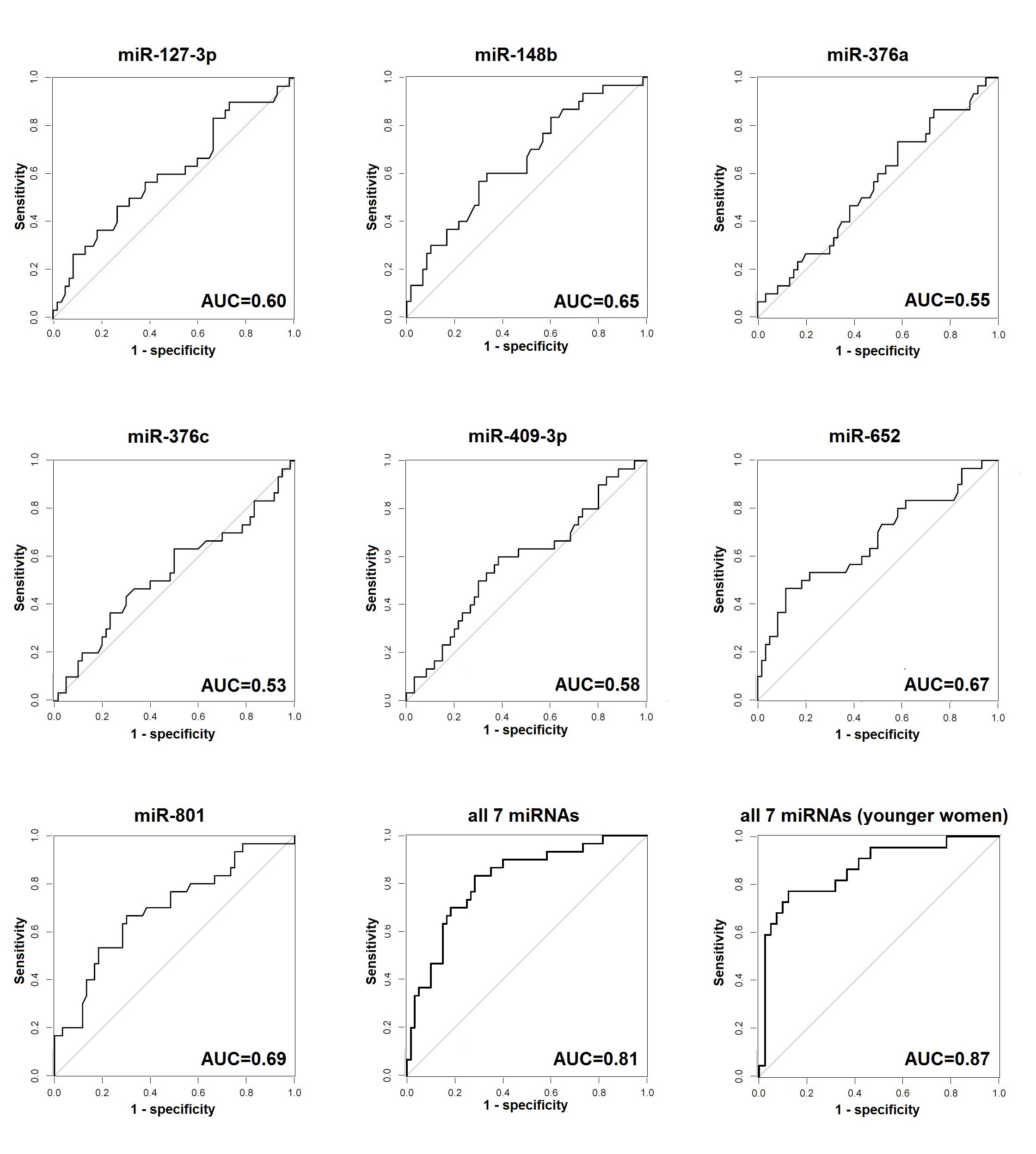

Supplement: Figure S3 — The diagnostic potential of circulating miRNAs for benign breast tumors (independent validation cohort B). In ROC curve analysis individual circulating miRNAs had discriminatory accuracy of 0.53–0.69 for plasma samples derived from healthy women and those with benign breast tumors. The accuracy was good for circulating miR-148b, miR-652 and miR-801, which were found to be significantly elevated in the plasma of women with benign breast tumors when compared to healthy women, while it was somewhat poor for the other four miRNAs. A combination of all seven circulating miRNAs yielded the highest discriminatory power for the detection of benign tumors with an AUC equal to 0.81, which was superior for younger women up to 50 years of age, where the AUC reached 0.87. (TIF) [file pone.0076729.s003.tif]

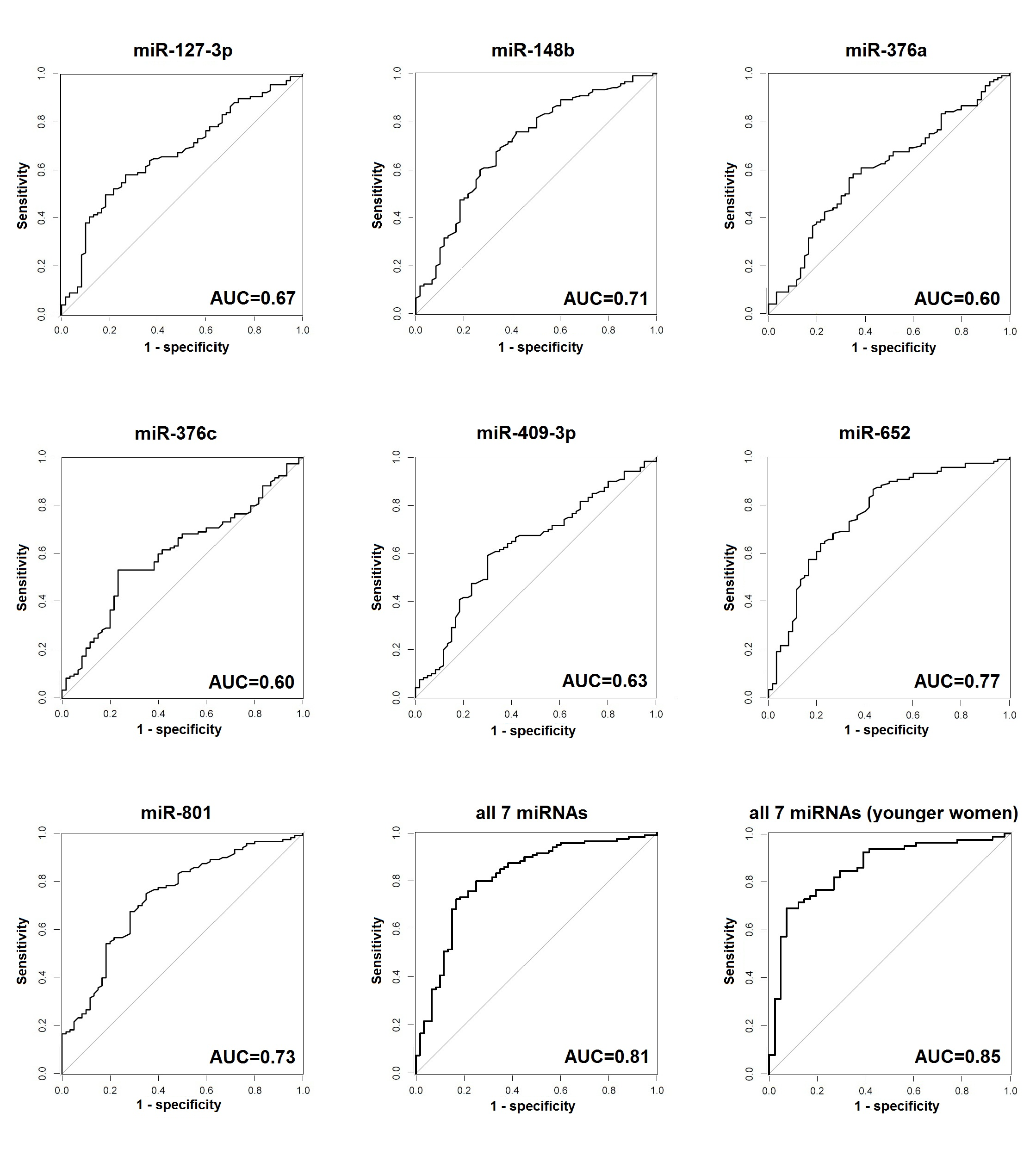

Supplement: Figure S4 — The diagnostic potential of circulating miRNAs for malignant breast tumors (independent validation cohort B). In ROC curve analysis individual circulating miRNAs were found to have discriminatory accuracy of 0.60–0.77 between plasma samples derived from healthy women and those with breast cancer. A combination of all seven circulating miRNAs yielded the highest discriminatory power for the detection of malignant breast tumors with an AUC equal to 0.81, which was even higher in younger women (≤50 years) with an AUC of 0.85. (TIF) [file pone.0076729.s004.tif]
