# Supplementary material for: Plasma MicroRNA Panel for Minimally Invasive Detection of Breast Cancer
Source: PLoS One. 2013 Oct 23;8(10):e76729. doi: 10.1371/journal.pone.0076729 (PMC3806790; doi:10.1371/journal.pone.0076729)
Supplement: Table S1 — Lifestyle data of healthy controls in validation cohorts A and B. (DOC) [file pone.0076729.s005.doc]

**Table S1.** **Lifestyle data of healthy controls in validation cohorts A and B.**

| **Characteristics** |  | **Cohort A**  **(n=80)** | **Cohort B**  **(n=60)** |
| --- | --- | --- | --- |
| **Age range (years):** |  | 28-63 | 29-77 |
| **Mean (median) age:** |  | 44.9 (45.0) | 46.5 (48.0) |
| **Body mass index (BMI)** | <20 | 9 | 6 |
|  | 20-25 | 46 | 29 |
|  | >25 | 23 | 24 |
|  | unknown | 2 | 1 |
| **Age of menarche** | <12 | 16 | 8 |
|  | 12-13 | 34 | 23 |
|  | >13 | 27 | 26 |
|  | unknown | 3 | 3 |
| **Gynecological diseases*** | yes | 30 | 23 |
|  | no | 48 | 36 |
|  | unknown | 2 | 1 |
| **Hormone intake in the past**** | yes | 65 | 53 |
|  | no | 12 | 6 |
|  | unknown | 3 | 1 |
| **Number of pregnancies** | 0 | 31 | 19 |
|  | 1 or more | 47 | 39 |
|  | unknown | 2 | 2 |
| **Smoking habits***** | has never smoked | 32 | 20 |
|  | former smoker | 20 | 22 |
|  | sporadic smoker | 15 | 10 |
|  | regular smoker | 11 | 6 |
|  | unknown | 2 | 2 |
| **Moderate alcohol intake****** | regularly | 28 | 24 |
|  | occassionally | 34 | 28 |
|  | never | 16 | 6 |
|  | unknown | 2 | 2 |
| **Physical excercise****** | regular | 54 | 52 |
|  | never | 24 | 6 |
|  | unknown | 2 | 2 |

* endometriosis, myoma, ovarian cyst and polycystic ovary syndrome

** hormone intake at any time in the past (including contraception pills and drugs prescribed for menstrual disorders or acne)

*** smokers smoke(d) cigarettes on a regular basis (at least 1 cigarette a day for at least 1 year) and sporadic smokers only occasionally (but not more than 5-10 a month)

**** regular includes daily or weekly alcohol consumption/physical exercise
