# Supplementary material for: Plasma MicroRNA Panel for Minimally Invasive Detection of Breast Cancer
Source: PLoS One. 2013 Oct 23;8(10):e76729. doi: 10.1371/journal.pone.0076729 (PMC3806790; doi:10.1371/journal.pone.0076729)
Supplement: Table S3 — Circulating miRNAs deregulated in the plasma of early stage breast cancer cases compared to healthy controls in TLDA array re-analysis. All candidate markers we analyzed are in bold, the three new marker candidates chosen for validation are underlined and the finally validated miRNAs are additionally italicized. (DOC) [file pone.0076729.s007.doc]

**Table S3.** **Circulating miRNAs deregulated in the plasma of early stage breast cancer cases compared to healthy controls in TLDA array re-analysis.** All candidate markers we analyzed are in bold, the three new marker candidates chosen for validation are underlined and the finally validated miRNAs are additionally italicized.

| **miRNA** | **P value** | **adj. P value (FDR)** | **mean Ct (controls)** | **mean Ct (cases)** | **ΔCt*** |
| --- | --- | --- | --- | --- | --- |
| ***hsa-miR-148b*** | 0,0005 | 0,0647 | 31,6 | 30,4 | 1,2 |
| hsa-miR-328 | 0,0004 | 0,0647 | 28,6 | 27,9 | 0,7 |
| ***hsa-miR-376c*** | 0,0002 | 0,0647 | 32,0 | 30,4 | 1,6 |
| ***hsa-miR-652*** | 0,0008 | 0,0785 | 31,9 | 30,7 | 1,2 |
| hsa-miR-320 | 0,0013 | 0,1073 | 24,0 | 24,8 | -0,8 |
| hsa-miR-145 | 0,0039 | 0,2252 | 28,1 | 27,1 | 1,0 |
| hsa-miR-339-3p | 0,0038 | 0,2252 | 30,9 | 30,2 | 0,7 |
| hsa-miR-193a-3p | 0,0067 | 0,2789 | 38,3 | 40,0 | -1,7 |
| **hsa-miR-206** | 0,0069 | 0,2789 | 29,9 | 31,7 | -1,8 |
| ***hsa-miR-801*** | 0,0066 | 0,2789 | 30,6 | 28,4 | 2,2 |
| **hsa-miR-139-3p** | 0,010 | 0,3768 | 29,8 | 34,9 | -5,1 |
| hsa-miR-221 | 0,015 | 0,3813 | 28,2 | 27,7 | 0,5 |
| ***hsa-miR-376a*** | 0,014 | 0,3813 | 32,9 | 31,5 | 1,4 |
| hsa-miR-138-1* | 0,013 | 0,3813 | 28,0 | 28,7 | -0,7 |
| hsa-miR-190b | 0,015 | 0,3813 | 33,9 | 32,9 | 1,0 |
| ***hsa-miR-409-3p*** | 0,013 | 0,3813 | 34,0 | 32,3 | 1,7 |
| hsa-miR-424 | 0,016 | 0,3899 | 38,3 | 35,3 | 3,0 |
| hsa-miR-184 | 0,020 | 0,4170 | 39,4 | 36,7 | 2,7 |
| hsa-miR-875-5p | 0,019 | 0,4170 | 34,0 | 33,1 | 0,9 |
| hsa-miR-93* | 0,024 | 0,4596 | 30,2 | 31,2 | -1,0 |
| hsa-miR-526b* | 0,024 | 0,4596 | 36,6 | 38,6 | -2,0 |
| let-7c | 0,036 | 0,5168 | 30,0 | 30,6 | -0,6 |
| hsa-miR-18a | 0,038 | 0,5168 | 29,2 | 28,5 | 0,7 |
| hsa-miR-29a | 0,049 | 0,5168 | 26,8 | 27,3 | -0,5 |
| hsa-miR-29c | 0,043 | 0,5168 | 30,3 | 30,8 | -0,5 |
| ***hsa-miR-127-3p*** | 0,047 | 0,5168 | 33,1 | 31,2 | 1,9 |
| hsa-miR-190 | 0,042 | 0,5168 | 36,4 | 34,2 | 2,2 |
| hsa-miR-323-3p | 0,049 | 0,5168 | 31,0 | 30,5 | 0,5 |
| hsa-miR-485-3p | 0,040 | 0,5168 | 32,0 | 31,3 | 0,7 |
| hsa-miR-519a | 0,031 | 0,5168 | 35,7 | 38,1 | -2,4 |
| hsa-miR-548d-3p | 0,040 | 0,5168 | 40,0 | 38,8 | 1,2 |
| hsa-miR-579 | 0,035 | 0,5168 | 32,3 | 33,1 | -0,8 |
| hsa-miR-598 | 0,041 | 0,5168 | 31,4 | 32,0 | -0,6 |
| hsa-miR-200a* | 0,042 | 0,5168 | 37,6 | 40,0 | -2,4 |
| hsa-miR-148b* | 0,034 | 0,5168 | 38,8 | 36,9 | 1,9 |
| hsa-miR-34a* | 0,049 | 0,5168 | 31,3 | 32,3 | -1,0 |
| hsa-miR-941 | 0,042 | 0,5168 | 37,8 | 40,0 | -2,2 |
| hsa-miR-188-5p | 0,047 | 0,5168 | 28,7 | 29,2 | -0,5 |

* ΔCt = mean Ct_controls_ - mean Ct_cases_
