# Supplementary material for: Plasma MicroRNA Panel for Minimally Invasive Detection of Breast Cancer
Source: PLoS One. 2013 Oct 23;8(10):e76729. doi: 10.1371/journal.pone.0076729 (PMC3806790; doi:10.1371/journal.pone.0076729)
Supplement: Table S4 — Circulating miRNAs deregulated in the plasma of early stage breast cancer cases (stage I and/or stage II) compared to healthy controls. In the validation cohorts circulating miR-127-3p, miR-148b, miR-409-3p, miR-652 and miR-801 were significantly elevated even in stage I and/or stage II breast cancer patients compared to healthy controls. A two-tailed P<0.05 was considered statistically significant (Wilcoxon rank sum test). Ctrls = controls. (DOC) [file pone.0076729.s008.doc]

**Table S4.** **Circulating miRNAs deregulated in the plasma of early stage breast cancer cases (stage I and/or stage II) compared to healthy controls.** In the validation cohorts circulating miR-127-3p, miR-148b, miR-409-3p, miR-652 and miR-801 were significantly elevated even in stage I and/or stage II breast cancer patients compared to healthy controls. A two-tailed P<0.05 was considered statistically significant (Wilcoxon rank sum test). Ctrls = controls.

| **Wilcoxon test**  **(P value)** | **First validation cohort (cohort A)** | | | | | | |
| --- | --- | --- | --- | --- | --- | --- | --- |
| **Comparison** | **miR-127-3p** | **miR-148b** | **miR-376a** | **miR-376c** | **miR-409-3p** | **miR-652** | **miR-801** |
| **Ctrls vs. stage I** | 0.046 | 0.02 | 0.13 | 0.005 | 0.009 | 0.003 | 0.18 |
| **Ctrls vs. stage II** | 0.0008 | 0.009 | 0.08 | 0.002 | 0.0008 | 0.01 | 0.0007 |
| **Wilcoxon test** | **Independent validation cohort (cohort B)** | | | | | | |
| **Comparison** | **miR-127-3p** | **miR-148b** | **miR-376a** | **miR-376c** | **miR-409-3p** | **miR-652** | **miR-801** |
| **Ctrls vs. stage I** | 0.007 | 0.0003 | 0.09 | 0.06 | 0.02 | < 0.0001 | 0.0002 |
| **Ctrls vs. stage II** | 0.02 | 0.002 | 0.23 | 0.30 | 0.08 | < 0.0001 | 0.0004 |
