# Supplementary material for: Plasma MicroRNA Panel for Minimally Invasive Detection of Breast Cancer
Source: PLoS One. 2013 Oct 23;8(10):e76729. doi: 10.1371/journal.pone.0076729 (PMC3806790; doi:10.1371/journal.pone.0076729)

**Table S5. Inter-correlations between miRNA levels in plasma.** Spearman rank correlation coefficients (ρ) between different circulating miRNAs with their 95% confidence intervals (CI) and P values.


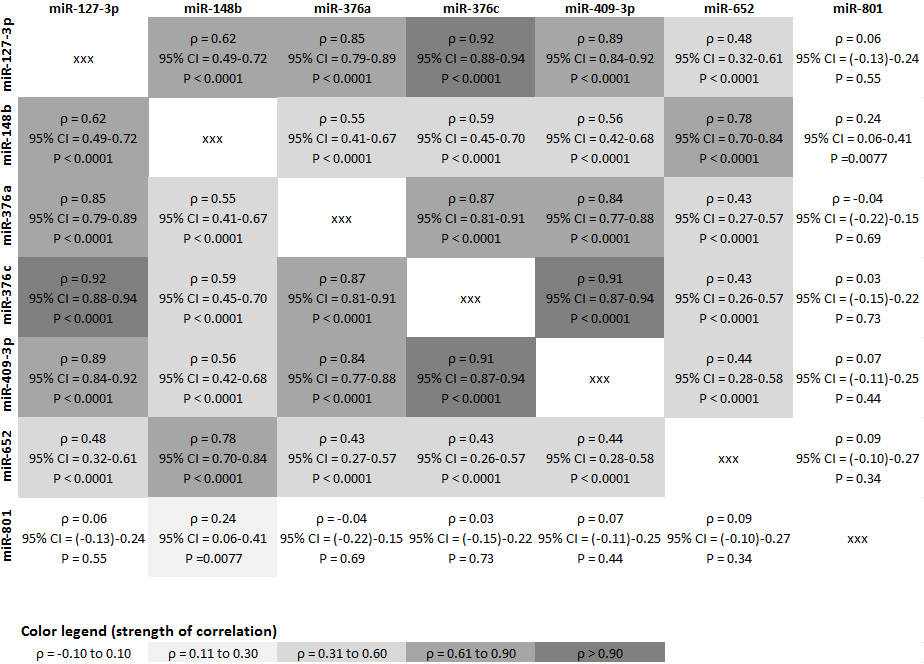

Supplement: Table S5 — Inter-correlations between miRNA levels in plasma. Spearman rank correlation coefficients (ρ) between different circulating miRNAs with their 95% confidence intervals (CI) and P values. (DOC) [file pone.0076729.s009.doc]
